# Sensitivity of Seven Diverse Species to Blue and Green Light: Interactions with Photon Flux

**DOI:** 10.1371/journal.pone.0163121

**Published:** 2016-10-05

**Authors:** M. Chase Snowden, Kevin R. Cope, Bruce Bugbee

**Affiliations:** Crop Physiology Laboratory, Department of Plants Soils and Climate, Utah State University, 4820 Old Main Hill, Logan, UT, 84322–4820, United States of America; University of Tsukuba, JAPAN

## Abstract

Despite decades of research, the effects of spectral quality on plant growth, and development are not well understood. Much of our current understanding comes from studies with daily integrated light levels that are less than 10% of summer sunlight thus making it difficult to characterize interactions between light quality and quantity. Several studies have reported that growth is increased under fluorescent lamps compared to mixtures of wavelengths from LEDs. Conclusions regarding the effect of green light fraction range from detrimental to beneficial. Here we report the effects of eight blue and green light fractions at two photosynthetic photon fluxes (PPF; 200 and 500 μmol m^-2^ s^-1^; with a daily light integral of 11.5 and 29 mol m^-2^ d^-1^) on growth (dry mass), leaf expansion, stem and petiole elongation, and whole-plant net assimilation of seven diverse plant species. The treatments included cool, neutral, and warm white LEDs, and combinations of blue, green and/or red LEDs. At the higher PPF (500), increasing blue light in increments from 11 to 28% reduced growth in tomato, cucumber, and pepper by 22, 26, and 14% respectively, but there was no statistically significant effect on radish, soybean, lettuce or wheat. At the lower PPF (200), increasing blue light reduced growth only in tomato (41%). The effects of blue light on growth were mediated by changes in leaf area and radiation capture, with minimal effects on whole-plant net-assimilation. In contrast to the significant effects of blue light, increasing green light in increments from 0 to 30% had a relatively small effect on growth, leaf area and net assimilation at either low or high PPF. Surprisingly, growth of three of the seven species was not reduced by a treatment with 93% green light compared to the broad spectrum treatments. Collectively, these results are consistent with a shade avoidance response associated with either low blue or high green light fractions.

## Introduction

Photobiology research began more than 200 years ago [1] with studies using primitive light sources and colored filters [2]. These early studies identified photoreceptors that determine aspects of plant development, and elucidated action spectra that determine photosynthetic quantum yield [3,4]. Improvements in lighting technology, particularly with the advent of light emitting diodes (LEDs) has facilitated the analysis of spectral responses, but interactions among species, light intensity, duration of the study, and other environmental parameters have reduced our ability to make broad photobiological conclusions for many whole-plant physiological responses.

### Effects of blue light on growth and development

Radiation provides energy for photosynthesis and information for photomorphogenesis [5]. Although red light (RL) efficiently drives photosynthesis, some blue light (BL) is typically necessary to improve growth (dry mass gain) and minimize shade avoidance responses including excessively elongated stems [6–8]. Yorio et al. [7] found that dry mass of spinach, radish and lettuce increased with the addition of 10% BL. Goins et al. [9] used identical light treatments to Yorio et al. [7] and found that dry mass of wheat with 10% BL was comparable to a white light control (33% BL). These results suggest that BL responses may be species dependent, but the effects of red/blue ratios and interactions with intensity remain unclear. Too much BL can inhibit growth. Recent studies have examined a range of BL fractions and found highest dry mass for lettuce, radish and pepper between about 5 and 15% BL. Cope and Bugbee [10] and Cope et al. [11] found that dry mass and leaf area decreased above 15% BL for lettuce, radish and pepper. Hernández and Kubota [12] reported decreased dry mass and leaf area for cucumber when the BL fraction increased above 10% with a pure RL background. In a greenhouse study, Hernández and Kubota [13] found that dry mass and leaf area of cucumber decreased with increasing BL. Wang et al. [14] found that lettuce shoot dry mass steadily decreased as BL increased from 8 to 50%. Dougher and Bugbee [15] described histological effects of BL on development of lettuce and soybean and found that increased BL decreased cell expansion and division in the stems and leaves of soybean. Interestingly, Son and Oh [16] reported the highest fresh and dry mass of lettuce at 0% BL (pure red light), but the plants were chlorotic and etiolated. With the exception of Son and Oh [16], these recent studies confirm early studies by Bula et al. [17] and Hoenecke et al. [18] who found that it was necessary to supplement red LEDs with blue fluorescent light to achieve highest dry mass in lettuce. Chen et al. [19] studied the addition of red and blue LEDs to a fluorescent light source. Supplementing fluorescent lamps with either RL or BL generally increased lettuce dry mass at the same photosynthetic photon flux (PPF), but the studies were conducted at a PPF of only 135 μmol m^-2^ s^-1^, which resulted in unusually slow growth in all treatments.

### Effects of spectral quality on photosynthesis in single leaves

The classic studies of McCree [3] and Inada [20] demonstrated that single leaves in narrow band light have a 25 to 35% higher quantum efficiency under RL (600 to 700 nm) than under BL (400 to 500 nm); and RL is 5 to 30% more efficient than GL (500 to 600 nm). It is important to remember that both of these studies were conducted on single leaves in low-light. In spite of this limitation, the relative quantum efficiency curves from these studies have often been used to predict spectral effects on whole-plant growth in long-term studies. Extrapolating from measurements on single leaves to whole plants or plant communities should be done with caution because changing light quality alters stem, petiole, and leaf expansion rates, which alter leaf area index and radiation capture.

### Effects of blue light on photosynthetic efficiency

In contrast to the studies by McCree [3] and Inada [20], which showed that RL was photosynthetically more efficient than BL, several studies have found that an increasing fraction of BL can increase photosynthetic capacity and efficiency. Goins et al. [9] were one of the first studies to report that BL increased single-leaf photosynthesis compared to RL alone in wheat. Hogewoning et al. [21] found that only 7% BL doubled the photosynthetic capacity (photosynthetic potential in higher PPF) over RL alone, and that photosynthetic capacity continued to increase with increasing BL up to 50% BL for cucumber. Terfa et al. [22] compared LEDs with 20% BL to high pressure sodium lamps with 5% BL and found that increased BL increased leaf thickness and photosynthetic capacity, but it is important to note that there was no effect of BL on whole plant dry mass. Wang et al. [23] compared BL effects in extremely low PPF (50 μmol m^-2^ s^-1^) and found similar photosynthetic rates in blue and white light, but the white light resulted in the highest dry mass per plant. In contrast to these studies, Ouzounis et al. [24] and Ouzounis et al. [25] showed decreased or no effect on photosynthesis with increasing BL for roses, chrysanthemums, campanulas and lettuce. The effect of BL on photosynthesis may vary with species, daily light integral, stage of development, and fraction of BL.

### Effects of green light

More than a half-century ago, after efficient fluorescent lamps became widely available, Frits Went lead a group of plant physiologists at the world-famous Cal Tech phytotron. This work used electric lights in controlled environments to elucidate numerous plant/environment interactions. Their wide-ranging studies were summarized by Went in a fascinating book, “*The experimental control of plant growth*” [26]. In one experiment, Went used colored filters below fluorescent lights to study the effect of GL. The studies were only six days long, but the results led Went to conclude that broad spectrum white light (with a high GL fraction) was less effective than RL and BL in the growth of tomatoes. The results of this study were consistent with expectations based on the minimal absorption of GL by chlorophyll (as shown in plant physiology textbooks at the time) and likely led to a paradigm of ineffective GL that has been accepted by plant biologists for 60 years.

Chlorophyll has minimal absorption of GL and there is a widespread perception that GL is not used efficiently in photosynthesis. Older editions of plant physiology textbooks regularly include chlorophyll a and b absorption curves and imply that GL is significantly less effective than RL and BL in driving photosynthesis. More recent editions of plant physiology textbooks (e.g. Taiz et al. [27]) now include comprehensive lists of photosynthetic pigments, including the GL absorbing pigment phycoerythrobilin. Sepúlveda-Ugarte et al. [28] showed that this GL absorbing pigment efficiently channeled excitation energy to chlorophyll a in *Gracilaria chilensis*, a red macroalga. It is clear that GL can be as effective as blue and red light in some species.

Green light penetrates deeper into leaves and deeper into canopies. Sun et al. [29] found that RL and BL drive CO_2_ fixation primarily in the upper palisade mesophyll while GL drives CO_2_ fixation in the lower palisade. Once the upper part of individual leaves and the upper canopy are saturated by RL and BL, additional GL should be beneficial in increasing whole plant photosynthesis [30]. Indeed, Terashima et al. [31] found that GL increased single leaf photosynthesis more than RL or BL at high PPF. In contrast to measurements of photosynthesis in single leaves at low light, a higher fraction of GL has the potential to increase whole-plant photosynthesis both in the bottom of upper leaves and by transmission to lower leaves.

Some long-term studies have found that dry mass increases with increasing GL fraction. Kim et al. [32] found that increasing GL from zero to 24% increased lettuce dry mass. Lin et al. [33] reported that lettuce grown without GL had reduced DM compared to two types of broad spectrum light sources (red + blue + white LEDs and fluorescent lamps) at the same PPF. Johkan et al. [34] studied lettuce at three PPFs (100, 200, and 300 μmol m^-2^ s^-1^) using three wavelengths of green LEDs and cool white fluorescent controls at all three PPFs and reported that the growth response to GL was inconsistent. Their results, however, indicate that dry mass decreased as PPF increased, so other unidentified environmental factors likely limited plant response to PPF. Other studies have found no effect of GL. In a recent study, Hernández and Kubota [12] found that increasing GL had no effect on the growth rate of cucumbers.

In a review of GL photoreceptors and responses, Folta and Maruhnich [35] suggested that increasing GL is “a signal to slow down or stop”, but they acknowledge that some species thrive in the green-enriched spectral environment at the bottom of plant communities. For this reason, the role of GL is thought to be especially important in the low light conditions that typically occur below plant canopies [36].

Differences among species and among studies have made it difficult to make general conclusions regarding GL effects. The effect of increasing GL on growth and development has become an increasingly important question as LED lighting evolves away from electrically-efficient purple fixtures (blue and red with no green) to fixtures with white LEDs that have a high fraction of GL.

### The beneficial effects of fluorescent light: direct vs. diffuse light

Several studies have reported increased growth under fluorescent lamps compared to combinations of LEDs at the same PPF [7,9,19,23,33]. This increased growth has been interpreted as an indication that broad spectrum light is superior to combinations of narrow spectrum LEDs. Several other factors, however, can contribute to differences in growth. Fluorescent lamps have a higher fraction of diffuse light compared to the direct beam light from typical LEDs. Measurements and models of canopy photosynthesis have shown that diffuse light penetrates canopies better than direct light and that this increased penetration causes increased photosynthesis and dry mass [37–39]. Fluorescent lamps also have increased long-wave radiation, which warms leaves about 2°C more than LEDs at the same PPF [40]. Warmer leaves typically have increased leaf expansion rates and thus increased radiation capture. Fluorescent lamps also have some far-red radiation, which can cause a shade avoidance response and increase leaf and petiole expansion and thus radiation capture. For these reasons, it is inappropriate to interpret the increased growth under fluorescent lamps as a response to blue or green light.

### Short-term single-leaf photosynthesis vs. long-term whole-plant assimilation

Most studies have used single-leaf techniques (gas-exchange or chlorophyll fluorescence [41]) to determine short-term photosynthetic efficiency. An alternative to short term measurements is to determine whole-plant net assimilation rate using crop growth analysis [42–45]. Crop growth analysis separates crop growth rate (CGR) into its two components: net assimilation rate (NAR) and leaf area index (LAI):

CGR=NAR×LAI

rearranging yields:

NAR=CGR÷LAI,

where CGR is in grams of dry mass m^-2^ d^-1^; NAR is in grams of dry mass m^-2^ d^-1^ of leaf; and LAI is in m^2^ of leaf per m^2^ of ground. The ratio of crop growth to leaf area index provides a measure of net assimilation integrated over time. This time interval is often 7 days, but it can be longer. When the photon flux is constant, this is a measure of photosynthetic efficiency.

The use of growth analysis in field studies typically shows that LAI and radiation capture are more closely related to growth than net assimilation. Poorter and Remkes [46] used growth analysis to analyze 24 wild species and found that net assimilation rate was relatively constant among species and that LAI better indicated competitive differences. Bullock et al. [47] studied spacing patterns in corn and found that yield increases were due to higher LAI with minimal change in net assimilation rate. In a controlled-environment, Klassen et al. [48] showed that daily carbon gain from canopy gas-exchange measurements was determined by LAI and light interception, with a constant NAR. Goins et al. [49] studied wavelengths of RL from 660 to 725 nm at a constant 8% BL level and found that increased far-red radiation led to increased leaf expansion, LAI and radiation capture, which caused increased growth, with no effect of leaf photosynthetic rate. Hogewoning et al. [50] reported that growth of cucumber was significantly greater under an artificial solar source that had significant far-red radiation compared to a control without far-red radiation. They attributed increased growth to increased light interception, not photosynthesis.

We sought to determine the effects of blue and green light on growth, leaf area development, whole-plant net assimilation, and carbon partitioning at two PPFs for seven diverse species. The arrangement of treatments facilitated the analysis of interactions between light quality and quantity across species.

## Materials and Methods

### Light treatments

**Fig 1:**
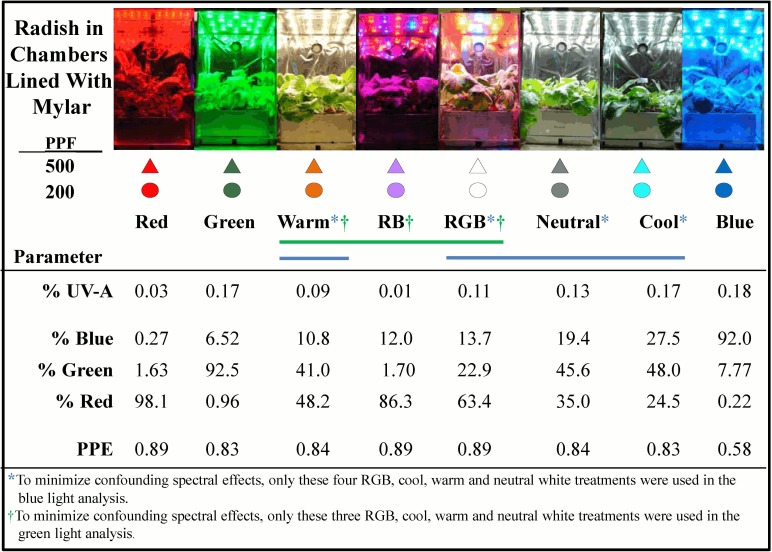
The eight spectral treatments and characteristics. Blue, green and red values are percent of total PPF (400 to 700 nm). UV-A is percent of total PPF. Phytochrome photoequilibrium (PPE) was determined as described by Sager et al. (1988). Symbols correspond to the color for each treatment and shape represents the two PPFs (200 and 500 μmol m^-2^ s^-1^), which are associated with DLIs of 11.5 and 29 mol m^-2^ d^-1^. Symbol shape and color are consistent in all figures.

**Fig 2:**
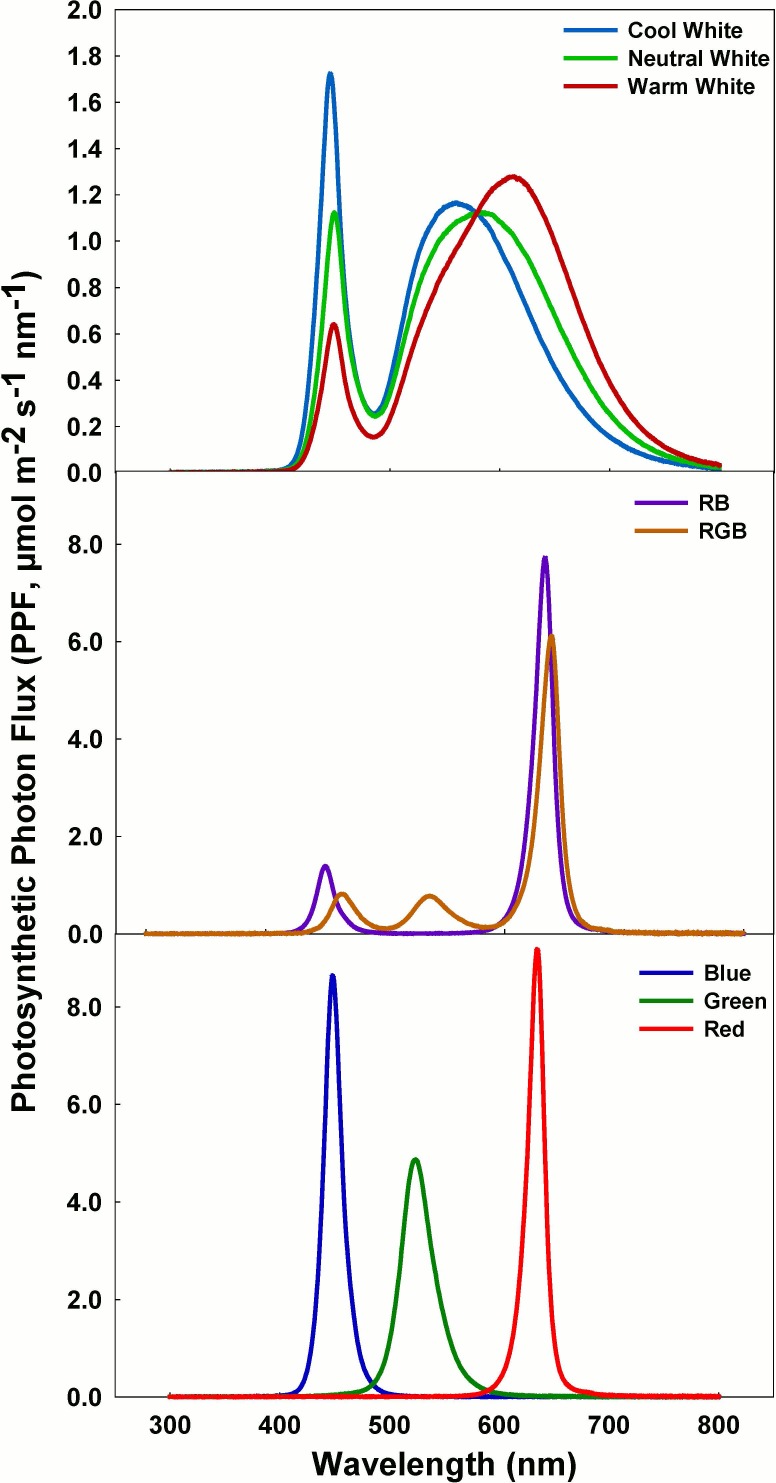
Spectral distributions of all eight LED treatments. Treatments include three types of white LEDs (cool, warm and neutral), red + blue (RB), red + green + blue (RGB) LEDs, and the narrow band red, green, and blue LEDs. Variation in spectral distribution between the 200 and 500 μmol m^-2^ s^-1^ treatments was negligible.

The experimental system included 16 chambers with eight spectral treatments at two daily light integrals (DLI; 11.5 and 29 mol m^-2^ d^-1^; about 25 and 60% of summer sunlight on a clear day). These DLIs were accomplished by providing a constant PPF of 200 and 500 μmol m^-2^ s^-1^ over a 16-h photoperiod. The treatments were developed using LED arrays including: warm, neutral, and cool white (Multicomp; Newark, Gaffney, SC), narrow band green, blue and red, and combinations of red and blue (RB) and red, green, and blue (RGB) (Luxeon Rebel Tri-Star LEDs; Quadica Developments Inc., Ontario, Canada). Measurement of phytochrome photoequilibrium, and the fraction of blue (400 to 500 nm), green (500 to 600 nm) and red (600 to 700 nm) light in in each growth chamber were made using a spectroradiometer (model PS-200; Apogee Instruments, Logan UT) (Fig 1). The spectral trace of each treatment is shown in Fig 2. PPF was measured with the spectroradiometer, and checked every three days using a quantum sensor (LI-188B; LI-COR, Lincoln, NE) that was calibrated for each treatment against the spectroradiometer. PPF was maintained at exactly 200 or 500 μmol m^-2^ s^-1^ at the top of the plant canopy by adjusting the electrical current to each LED array.

### Plant material and cultural conditions

Lettuce (*Lactuca sativa*, cv. Waldmann’s Green), cucumber (*Cucumis sativa*, cv. Sweet Slice), wheat (*Triticum aestivium* L. cv. USU-Apogee), tomato (*Solanum lycopersicum*, cv. Early girl), soybean (*Glycine max*, cv. Hoyt) and radish (*Raphanus sativus*, cv. Cherry Belle) seeds were pre-germinated, selected for uniformity, and eight seeds were transplanted to root modules (15 x 18 x 9 cm, L x W x H; 2430 cm^3^), except wheat in which twelve seeds were transplanted. Root modules were filled with horticultural grade soilless medium (1 peat: 1 vermiculite by volume) and 5 g of uniformly-mixed slow-release fertilizer (1 to 2 month release Polyon® 16-6-13; 16–2.6–10.8 as elemental N-P-K). The media was watered to excess with a complete, dilute fertilizer solution (100 ppm N; Scotts® Peat-Lite, 21-5-20; EC = 100 mS per m), and allowed to passively drain. This fertilizer solution was applied as needed to maintain ample root-zone moisture (every two to three days). The slow-release fertilizer and nutrient solution maintained a near-optimal leachate electrical conductivity between 100 and 150 mS per m (1.0 to 1.5 millimhos per cm; 1 to 1.5 dS per m). To improve uniformity, root modules in each chamber were rotated 180° at each watering event (every two or three days). The photoperiod was 16 h day/8 h night.

Pepper (*Capsicum annum*, cv. California Wonder) seeds were pre-germinated in a germination box for 7 days, and two pre-germinated seeds with emerging radicles were transplanted into 8 x 8 x 7 cm pots (448 cm^3^). The pots were filled with soilless medium identical to that used for the other species with 1 g of slow-release fertilizer incorporated into each pot. The pots were watered to excess with the same dilute fertilizer solution and allowed to passively drain. After planting, the pots were placed in a growth chamber (130 x 56 x 108 cm; 0.79 m^3^) with a PPF of 300 μmol m^-2^ s^-1^ provided by cool white fluorescent lamps and day/night temperature of 25/20°C. After 33 days, 48 of the most uniform plants were randomly assigned to 16 groups of four plants and were transplanted into root modules with the same dimensions as used for the other five species.

After planting, each root module was randomly placed into one of the 16 growth chambers (19.5 x 23 x 30 cm; 13455 cm^3^) that were lined with high-reflectance Mylar (Fig 1). Seedlings were thinned after emergence to four uniform seedlings, which grew until harvest. Wheat was not thinned and neither were peppers after transplanting.

In addition to rigorous procedures to achieve uniform plant emergence in each chamber in each trial, all chambers were well-ventilated to ensure uniform temperature, CO_2_ and relative humidity. Average air temperature differences among chambers were less than 0.2°C. Relative humidity averaged 40% and varied less than 3% among chambers; CO_2_ averaged 430 μmol mol^-1^ and varied less than 10 μmol mol^-1^ among chambers.

### Plant measurements

All species were harvested a few days after canopy closure, which occurred 21 days after emergence for most species. Cucumber and pepper were grown for 16 and 54 days after emergence respectively. Leaf area was measured using a leaf area meter (model LI-3000; LI-COR, Lincoln, NE). Stems and leaves were separated and dried to a constant mass at 80°C for dry mass (DM) determination. Root mass was not measured. Chlorophyll was measured with an optical chlorophyll meter (model MC-100, Apogee Instruments, Logan, UT) that was calibrated for each species according to the method of Parry et al. [51].

From the above measurements, leaf area index, specific leaf area, and net assimilation (g of DM per m^2^ leaf area) were determined for all species.

### Statistical analysis

#### Selection of comparable treatments

To mitigate potentially confounding factors for the effect of BL, statistical analysis included only the four treatments (RGB, cool, warm and neutral white) that had comparable RL and GL (Fig 1). The RB treatment was not included due to the low GL with this treatment (0.71%) compared to 21.6, 37.8, 29.0 and 34.5% for the RGB, cool, warm and neutral white treatments respectively. The narrow band treatments (red, blue and green) were not included in the analysis due to the potentially confounding effects of lack of other wavelengths. These treatments, however, were included on all figures to indicate the response to narrow band light.

To mitigate potentially confounding factors for the effect of GL, statistical analysis included only the three treatments (RB, warm and RGB treatments) with comparable RL and BL (Fig 1). The treatments not included in the regression model were included on all figures to provide a reference to the responses to these treatments. An analysis was also conducted including the potentially confounding treatments. This increased the number of degrees of freedom in the analysis from 12 to 15 and from 9 to 15, but the statistical significance was minimally changed and the conclusions remained unchanged. Consistent with conservative statistical procedures, the potentially confounding treatments were not included in the analysis.

Three replicate studies were conducted for each species. Regression analysis for BL effects included only the four treatments with comparable RL and GL (Cool, Warm, Neutral, RBG) (Fig 1). Variables were separately analyzed for each species at both PPFs. Regression analysis for GL effects included only the three treatments with comparable red and blue light (RB, RGB and warm) (Fig 1). Regression analysis included 12 data points for BL (3 reps x 4 treatments); and 9 data points for GL (3 reps x 3 treatments). Statistical analysis at *p = 0*.*05* was conducted with the PROC-REG package in SAS (version 9.3; Cary, NC, USA).

## Results

**Fig 3:**
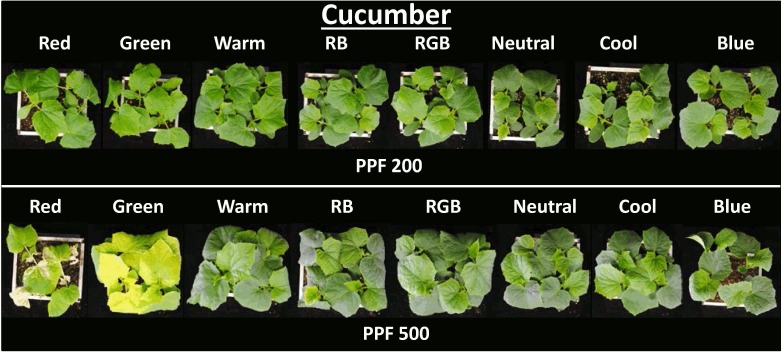
Overhead view of cucumber plants at harvest. Includes all eight LED treatments at both light intensities arranged from low to high BL fraction. There were four plants per treatment and three replicates per study. Note the difference in coloration with the green and red treatments at the 500 PPF level (DLI = 29 mol m^-2^ d^-1^).

An overhead view of one example replicate study with cucumber showing all eight treatments at both light levels just prior to harvest is provided in Fig 3. Each container with four plants is arranged in order of increasing BL from left to right. The chlorophyll concentration was dramatically reduced in the narrow band green treatment at the higher light level and this reduction occurred for many species. It is difficult to visually distinguish differences in leaf area among treatments at harvest because all treatments had reached canopy closure. However, narrow band BL at the high light level overall had a visually decreased leaf area, compared to the multi-wavelength treatments (RB, RGB, cool, neutral and warm white). These visual results suggest that differences among treatments were greater at the higher light level. Measurements of chlorophyll and leaf area were consistent with the visual observations.

### General comments relating to all figures

The order of presentation of species in the figures is based on sensitivity to BL. Tomato (at the top left in all figures) was generally the most sensitive and wheat (at the bottom in all figures) the least sensitive species. The regression line in this and all other figures includes only the three or four treatments that are directly comparable. All data points are included to facilitate the development of alternative hypotheses for the responses. Although the points are not connected, the effect of blue and green light may not be linear up to the high fractions of each color group.

### Dry mass

#### Effect of blue light

**Fig 4:**
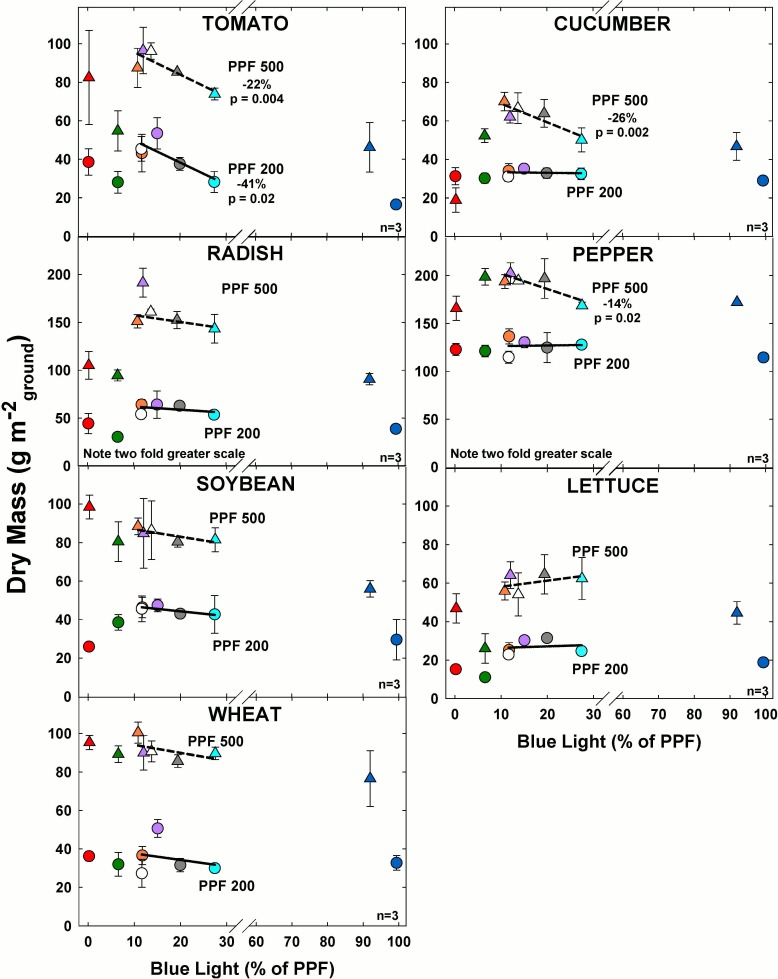
The effect of percent blue light on dry mass (DM) gain for seven species under two PPFs. Note scale break for percent BL between 30 and 60. Also note two-fold scale increase for DM in radish and pepper. Each data point shows the mean and standard deviation of three replicate studies for each species (n = 3). Some error bars are smaller than the symbol size. See Fig 1 for symbol color and shape legend. To minimize confounding spectral effects the regression line includes only the four treatments with comparable green and red wavelengths for each PPF. When significant, p-values and percent change are shown.

At the higher light level, dry mass (DM) decreased significantly in tomato, cucumber and pepper as BL increased (Fig 4). At the lower light level, tomato was the only species for which BL caused a significant decrease in DM.

As expected, DM increased with the 2.5 fold increase in PPF. For tomato, radish, soybean, lettuce and wheat, DM was nearly two and half times greater at high light level, but for cucumber and pepper, DM was only 40% greater at higher light level.

Overall the highest DM for all species tended to occur in the treatments with 11 to 15% BL and the effects of increasing BL were more pronounced at the higher light level.

#### Effect of green light

**Fig 5:**
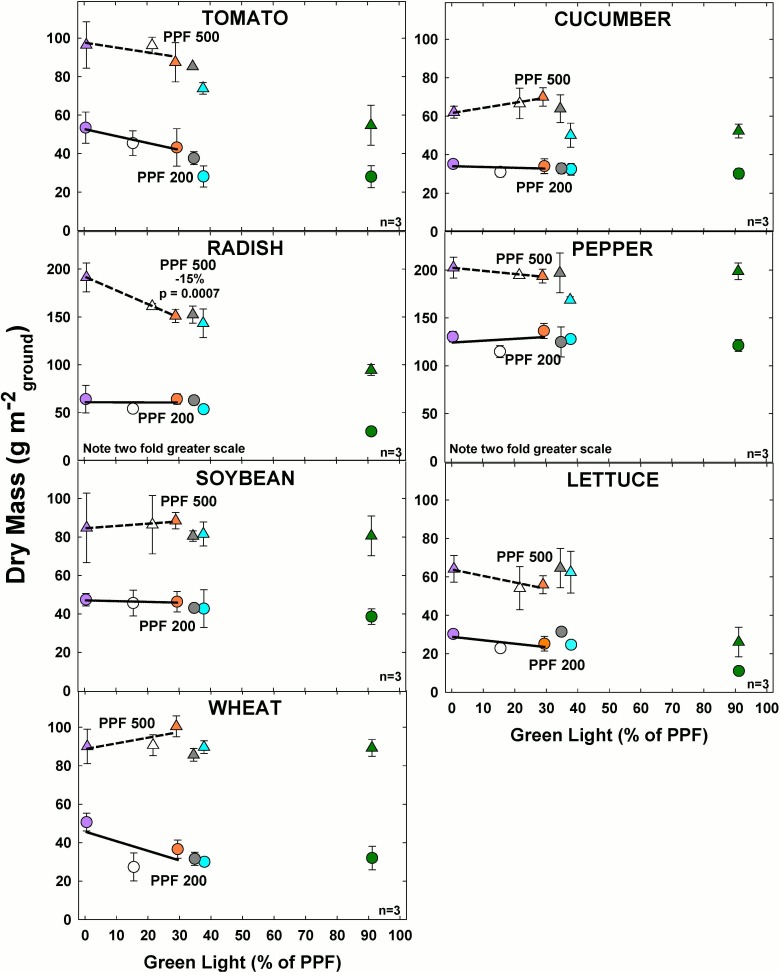
The effect of percent green light on dry mass (DM) gain for seven species under two PPFs. Note two-fold scale increase for DM in radish and pepper. Each data point shows the mean and standard deviation of three replicate studies for each species (n = 3). Some error bars are smaller than the symbol size. See Fig 1 for symbol color and shape legend. To minimize confounding spectral effects the regression line includes only the three treatments with comparable blue and red wavelengths for each PPF. When significant, p-values and percent change are shown.

There were no significant effects of GL on DM at the lower light level and there was minimal change in DM as GL increased from 0 to 30% at either light level (Fig 5). The only exception was radish, in which DM significantly decreased with increasing GL at the higher light level.

### Leaf area index

#### Effect of blue light

**Fig 6:**
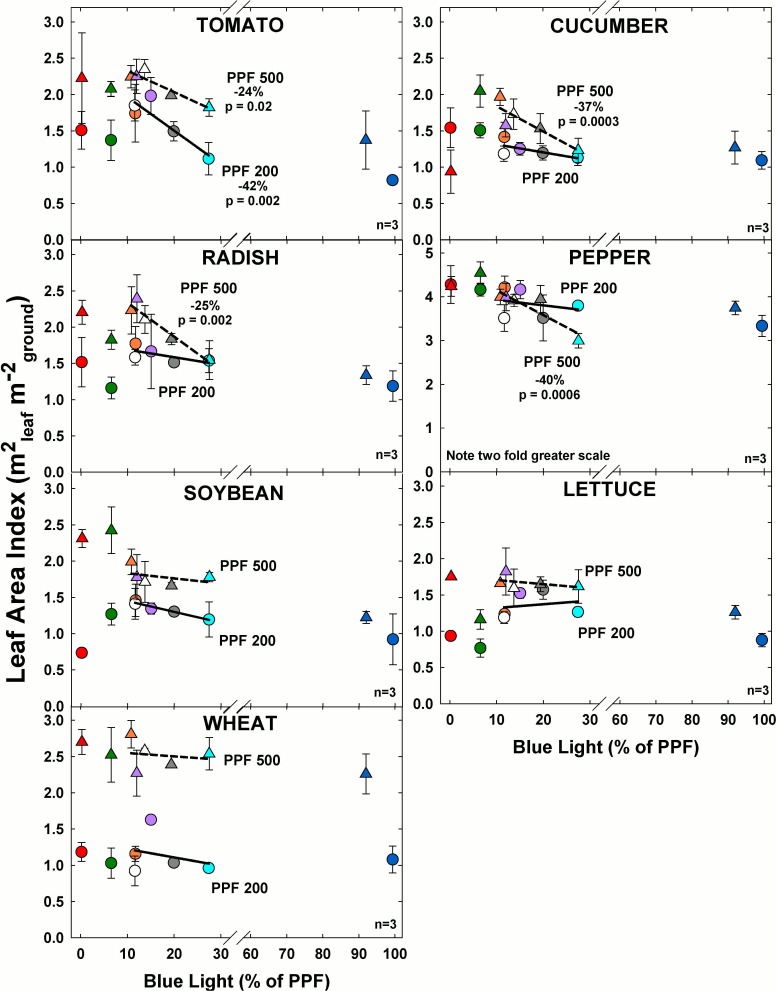
The effect of percent blue light on leaf area index (LAI) for seven species under two PPFs. Note scale break for percent BL between 30 and 60. Also note two fold scale increase for pepper. Each data point shows the mean and standard deviation of three replicate studies for each species (n = 3). Some error bars are smaller than the symbol size. See Fig 1 for symbol color and shape legend. To minimize confounding spectral effects the regression line includes only the four treatments with comparable green and red wavelengths for each PPF. When significant, p-values and percent change are shown.

Leaf area index (LAI) decreased significantly with increasing BL in tomato, cucumber, radish and pepper at the higher light level (Fig 6). At the lower light level, LAI significantly decreased with increasing BL only in tomato.

As expected, leaf area index increased with PPF. Similar to BL effects on DM, LAI tended to be higher in the treatments with the lower BL for all species at both light levels.

#### Effect of green light

**Fig 7:**
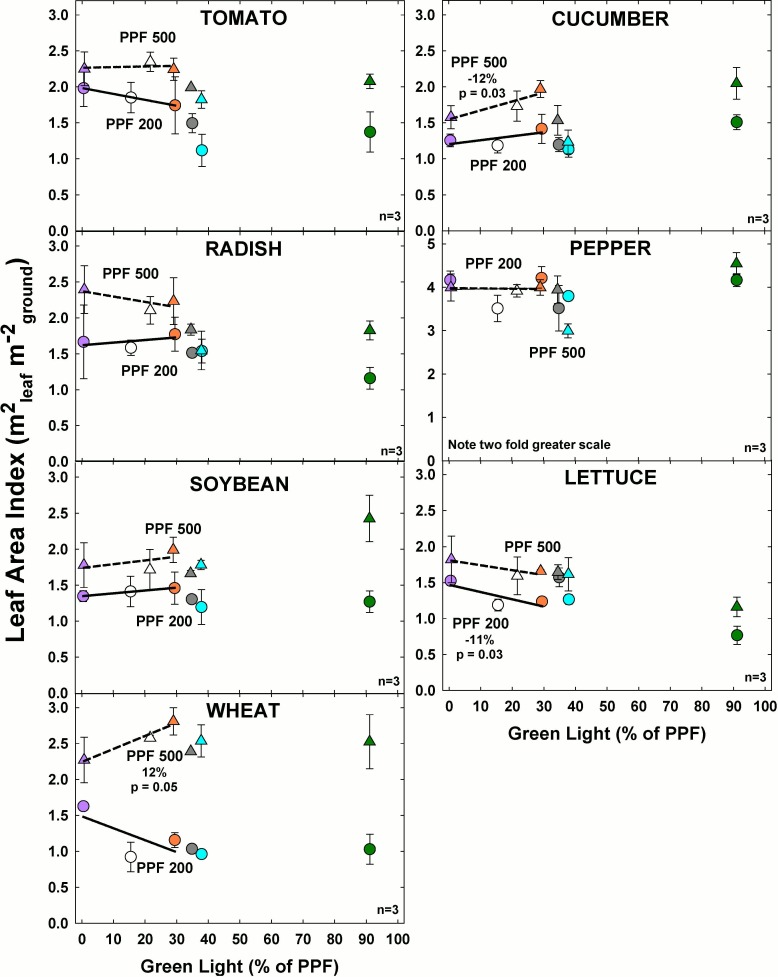
The effect of percent green light on leaf area index (LAI) for seven species under two PPFs. Note two fold scale increase for pepper. Each data point shows the mean and standard deviation of three replicate studies for each species (n = 3). Some error bars are smaller than the symbol size. See Fig 1 for symbol color and shape legend. To minimize confounding spectral effects the regression line includes only the three treatments with comparable blue and red wavelengths for each PPF. When significant, p-values and percent change are shown.

At the higher light level, LAI increased significantly with increasing GL only for cucumber and wheat (Fig 7). At the lower light level LAI decreased with increasing GL in lettuce. In all species except pepper, LAI increased with PPF.

### Net assimilation

#### Effect of blue light

**Fig 8:**
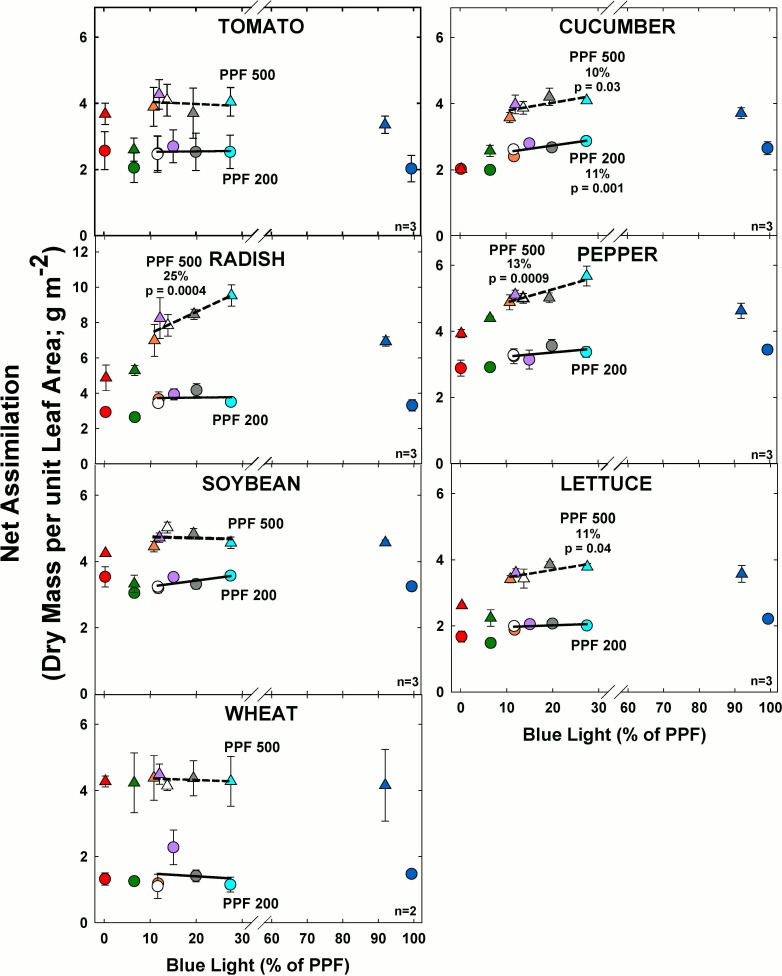
The effect of percent blue light on net assimilation for seven species under two PPFs. Note scale break for percent BL between 30 and 60. Each data point shows the mean and standard deviation of three replicate studies for each species (n = 3). Some error bars are smaller than the symbol size. See Fig 1 for symbol color and shape legend. To minimize confounding spectral effects the regression line includes only the four treatments with comparable green and red wavelengths for each PPF. When significant, p-values and percent change are shown.

At the higher light level, net assimilation significantly increased with increasing BL in cucumber, radish, pepper and lettuce (Fig 8). At the lower light level, net assimilation significantly increased with increasing BL only in cucumber and there was no significant effect on the other species. As expected, net assimilation greatly increased with PPF for all species.

#### Effect of green light

**Fig 9:**
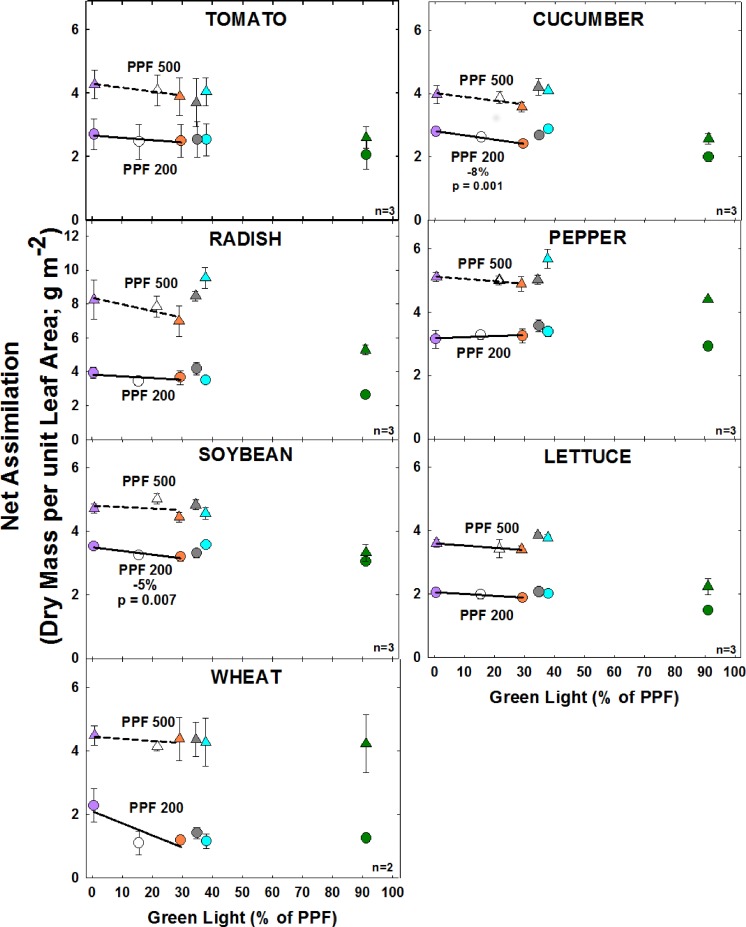
The effect of percent green light on net assimilation for seven species under two PPFs. Each data point shows the mean and standard deviation of three replicate studies for each species (n = 3). Some error bars are smaller than the symbol size. See Fig 1 for symbol color and shape legend. To minimize confounding spectral effects the regression line includes only the three treatments with comparable blue and red wavelengths for each PPF. When significant, p-values and percent change are shown. Regression line includes the RB, RBG and warm white treatments for each PPF. When significant, p-values and percent change are shown.

At the higher light level, there were no significant differences in net assimilation with increasing GL (Fig 9). At the lower light level, net assimilation significantly decreased with increasing GL for cucumber and soybean and there was no significant change for the other species.

Net assimilation greatly increased with PPF for each species. With the exception of pepper, which has an unusually high concentration of chlorophyll in its leaves, the highest net assimilation tended to occur in the treatments with the lowest GL.

### Stem length

#### Effect of blue light

**Fig 10:**
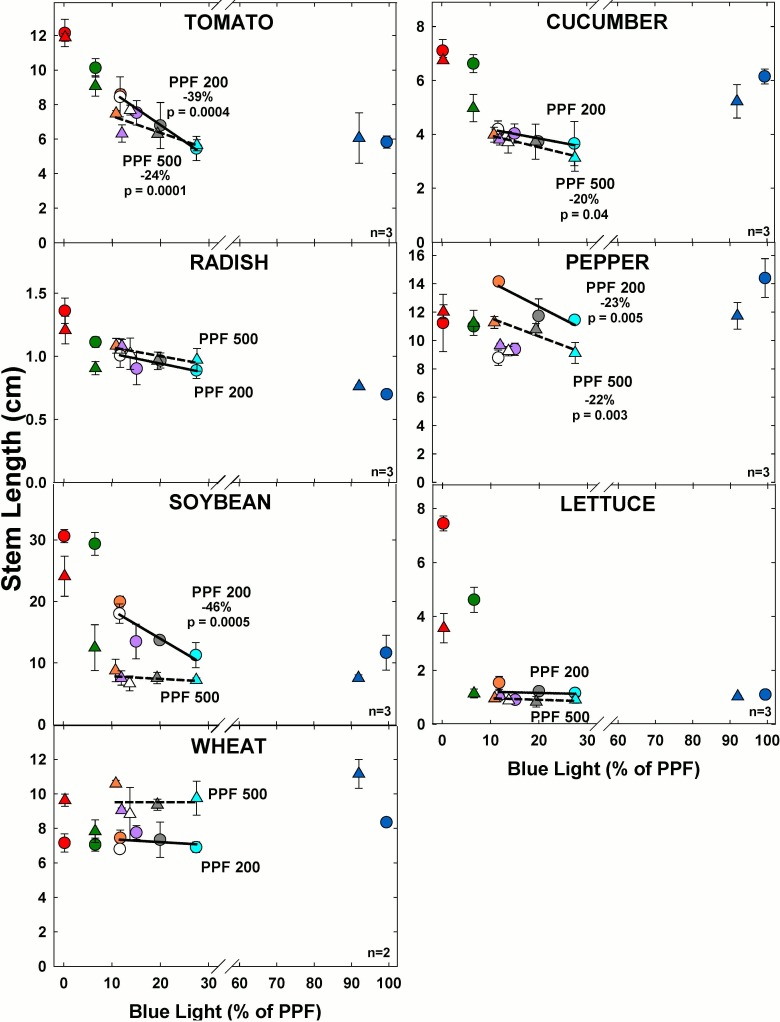
The effect of percent blue light on stem length for seven species under two PPFs. Note scale break for percent BL between 30 and 60. Each data point shows the mean and standard deviation of three replicate studies for each species (n = 3). Some error bars are smaller than the symbol size. See Fig 1 for symbol color and shape legend. To minimize confounding spectral effects the regression line includes only the four treatments with comparable green and red wavelengths for each PPF. When significant, p-values and percent change are shown.

At the higher light level, increasing BL significantly decreased stem length in tomato, cucumber, and pepper but there was no significant effect on the other species (Fig 10). At the lower light level, increasing BL significantly decreased stem length for tomato, pepper and soybean but there was no significant effect on the other species.

The higher PPF decreased stem length for pepper, soybean and increased stem length for wheat. Overall the longest stem length tended to occur in the treatments with lower BL.

#### Effect of green light

**Fig 11:**
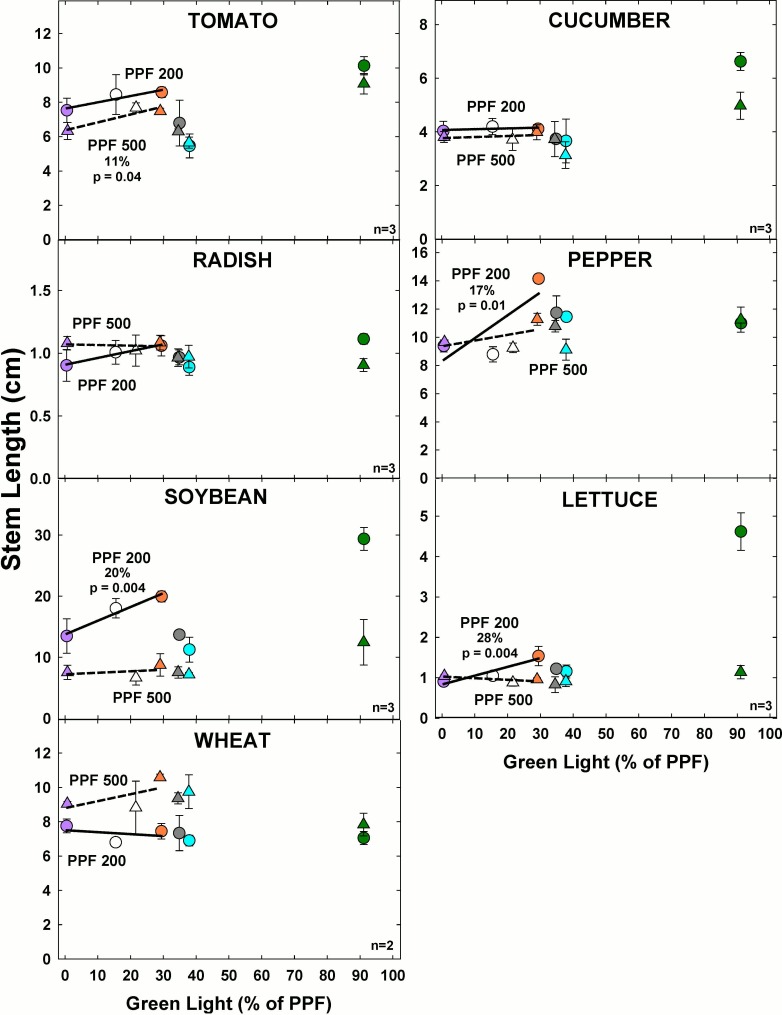
The effect of percent green light on stem length for seven species under two PPFs. Each data point shows the mean and standard deviation of three replicate studies for each species (n = 3). Some error bars are smaller than the symbol size. See Fig 1 for symbol color and shape legend. To minimize confounding spectral effects the regression line includes only the three treatments with comparable blue and red wavelengths for each PPF. When significant, p-values and percent change are shown.

At the higher light level, stem length significantly increased with increasing GL only for tomato (Fig 11). At the lower light level, stem length significantly increased with increasing GL for pepper, soybean and lettuce.

Stem length was similar between PPF levels for all species except soybean and wheat. Overall the longest stem length tended to occur in the treatments with higher GL fraction at both light levels.

### Petiole length

#### Effect of blue light

**Fig 12:**
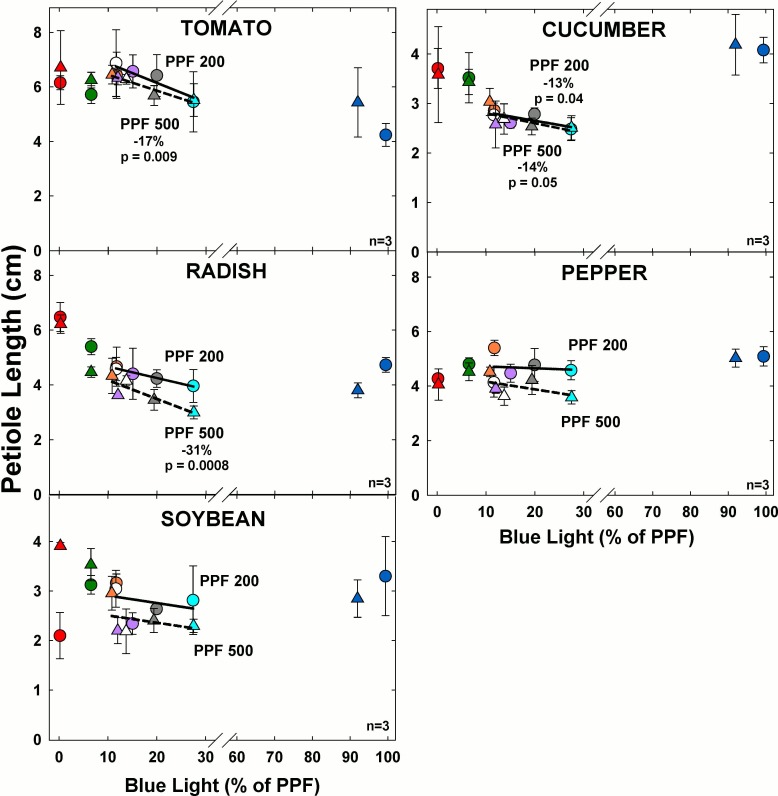
The effect of percent blue light on petiole length for seven species under two PPFs. Note scale break for percent BL between 30 and 60. Each data point shows the mean and standard deviation of three replicate studies for each species (n = 3). Some error bars are smaller than the symbol size. See Fig 1 for symbol color and shape legend. To minimize confounding spectral effects the regression line includes only the four treatments with comparable green and red wavelengths for each PPF. When significant, p-values and percent change are shown.

At the higher light level, petiole length significantly decreased with increasing BL for tomato, cucumber and radish (Fig 12). At the lower light level, petiole length significantly decreased with increasing BL only for cucumber but it tended to decrease for all species.

Petiole length increased at the lower PPF for all species except cucumber. This is a typical shade avoidance response and results in increased radiation capture in low light.

Lettuce and wheat are not included in these results because they do not develop measureable petioles.

#### Effect of green light

**Fig 13:**
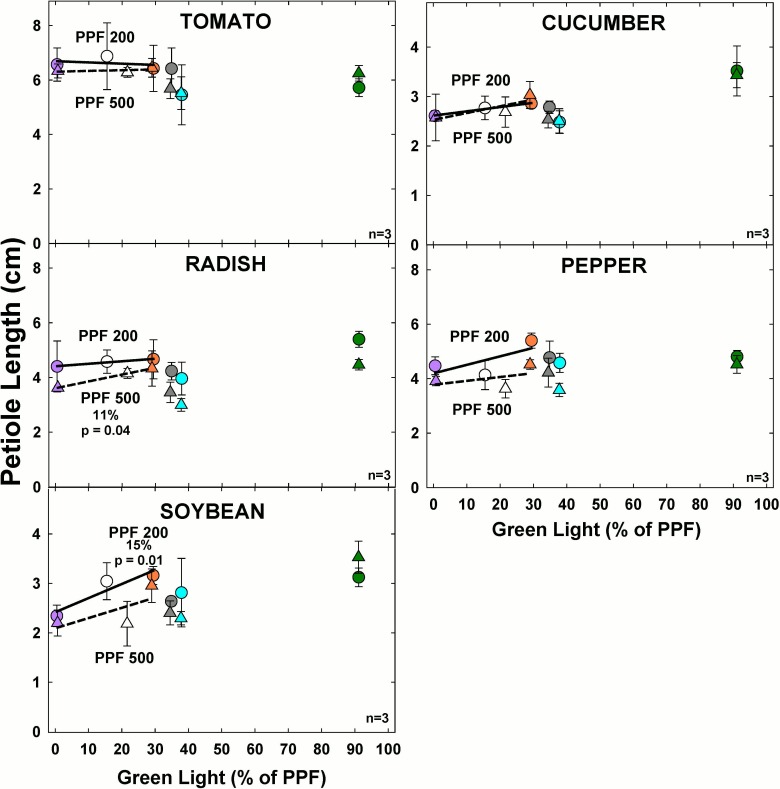
The effect of percent green light on petiole length for seven species under two PPFs. Each data point shows the mean and standard deviation of three replicate studies for each species (n = 3). Some error bars are smaller than the symbol size. See Fig 1 for symbol color and shape legend. To minimize confounding spectral effects the regression line includes only the three treatments with comparable blue and red wavelengths for each PPF. When significant, p-values and percent change are shown.

At the higher light level, increasing GL significantly increased petiole length only for radish (Fig 13). At the lower light level, increasing GL significantly increased petiole length only for soybean.

Petiole lengths were longer at the lower PPF in all species, likely because of a shade avoidance response. Overall, petioles tended to be longer with increasing GL but the effect was not always statistically significant.

### Specific leaf area

#### Effect of blue light

**Fig 14:**
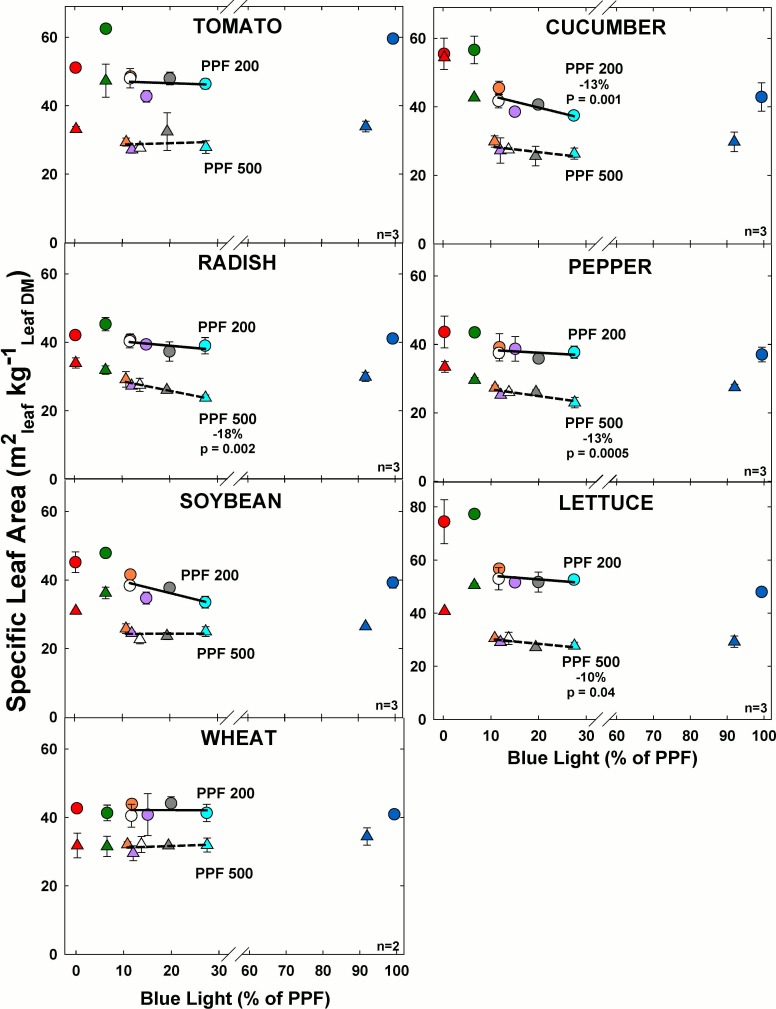
The effect of percent blue light on specific leaf area for seven species under two PPFs. Note scale break for percent BL between 30 and 60. Each data point shows the mean and standard deviation of three replicate studies for each species (n = 3). Some error bars are smaller than the symbol size. See Fig 1 for symbol color and shape legend. To minimize confounding spectral effects the regression line includes only the four treatments with comparable green and red wavelengths for each PPF. When significant, p-values and percent change are shown.

Specific leaf area significantly decreased (m^2^ per kg; thicker leaves) with increasing BL for radish, pepper and lettuce at the higher light level among comparable treatments (Fig 14). For all other species at the higher light level there was minimal change in specific leaf area as BL increased. At the lower light level, specific leaf area significantly decreased with increasing BL only for cucumber. For all other species at the lower light level there was minimal change in specific leaf area as BL increased.

Specific leaf area greatly decreased (thicker leaves) as PPF increased for each species. At lower light levels leaves are typically thinner to capture more light per unit leaf mass. Overall the highest specific leaf area (thinnest leaves) occurred in treatments with the least BL for all species at both light levels.

#### Effect of green light

**Fig 15:**
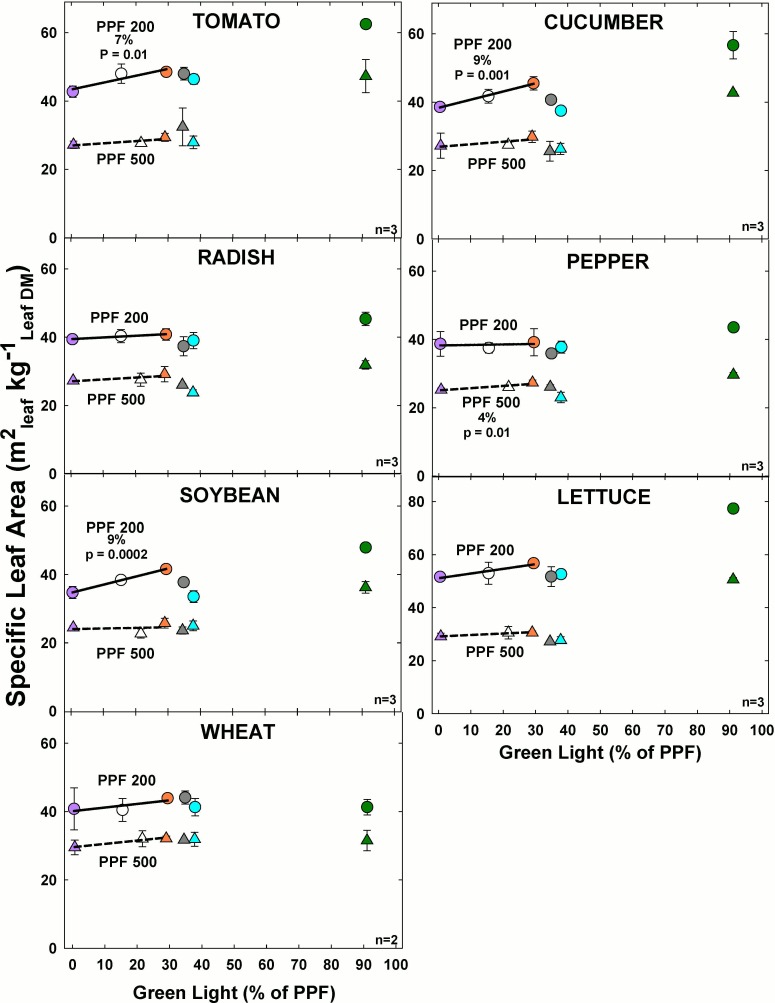
The effect of percent green light on specific leaf area for seven species under two PPFs. Each data point shows the mean and standard deviation of three replicate studies for each species (n = 3). Some error bars are smaller than the symbol size. See Fig 1 for symbol color and shape legend. To minimize confounding spectral effects the regression line includes only the three treatments with comparable blue and red wavelengths for each PPF. When significant, p-values and percent change are shown.

Specific leaf area significantly increased with increasing GL only for pepper at the higher light level among the comparable treatments (Fig 15). For all other species there was minimal change in specific leaf area as GL increased at the higher light level. At the lower light level, specific leaf area significantly increased with increasing GL for tomato, cucumber and soybean. For all other species at the lower light level there was minimal change in stem length as GL increased.

Specific leaf area greatly decreased as PPF increased for each species. This occurred as a result of leaves being thicker at the lower light level due to decreased intensity and slower growth. Overall the highest specific leaf area typically occurred in treatments with the most GL for all species at both light levels, except wheat.

### Chlorophyll

#### Effect of blue light

**Fig 16:**
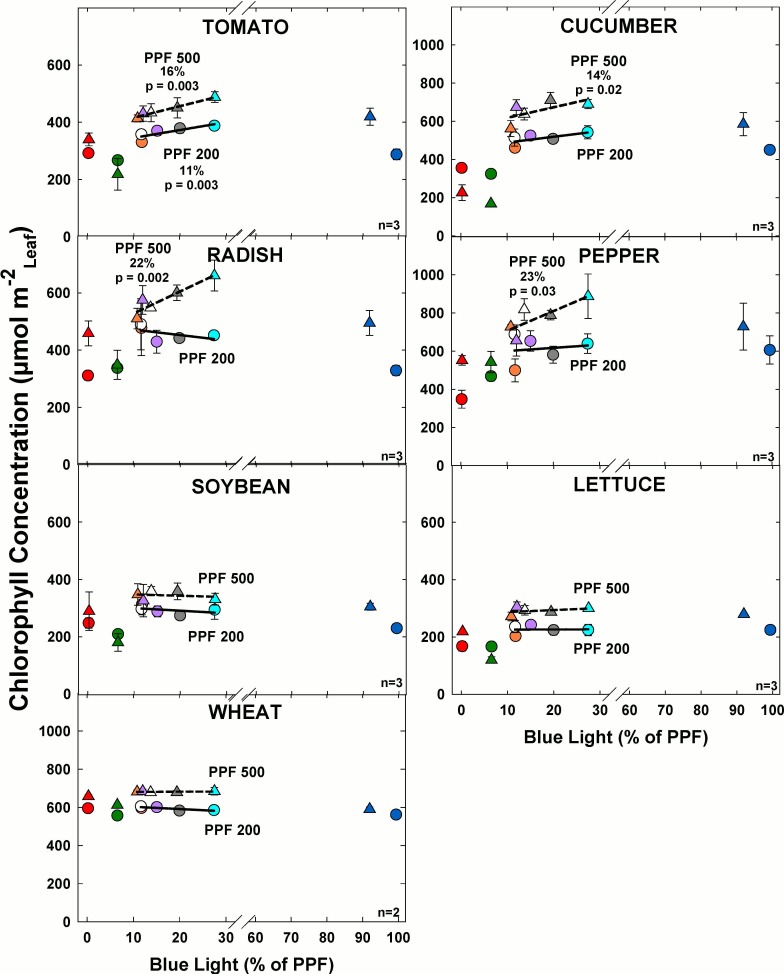
The effect of percent blue light on chlorophyll concentration for seven species under two PPFs. Note scale break for percent BL between 30 and 60. Each data point shows the mean and standard deviation of three replicate studies for each species (n = 3). Some error bars are smaller than the symbol size. See Fig 1 for symbol color and shape legend. To minimize confounding spectral effects the regression line includes only the four treatments with comparable green and red wavelengths for each PPF. When significant, p-values and percent change are shown.

Chlorophyll concentration significantly increased with increasing BL in tomato, cucumber, radish and pepper at the higher light level among comparable treatments (Fig 16). There was minimal change in chlorophyll concentration as BL increased for all other species at the higher light level. At the lower light level, chlorophyll concentration significantly increased with increasing BL only for tomato. For all other species at the lower light level, there was minimal change in chlorophyll concentration as BL increased.

Chlorophyll concentration increased with PPF. Overall the highest chlorophyll concentration at both light levels typically occurred in the treatments with 20 to 30% BL.

#### Effect of green light

**Fig 17:**
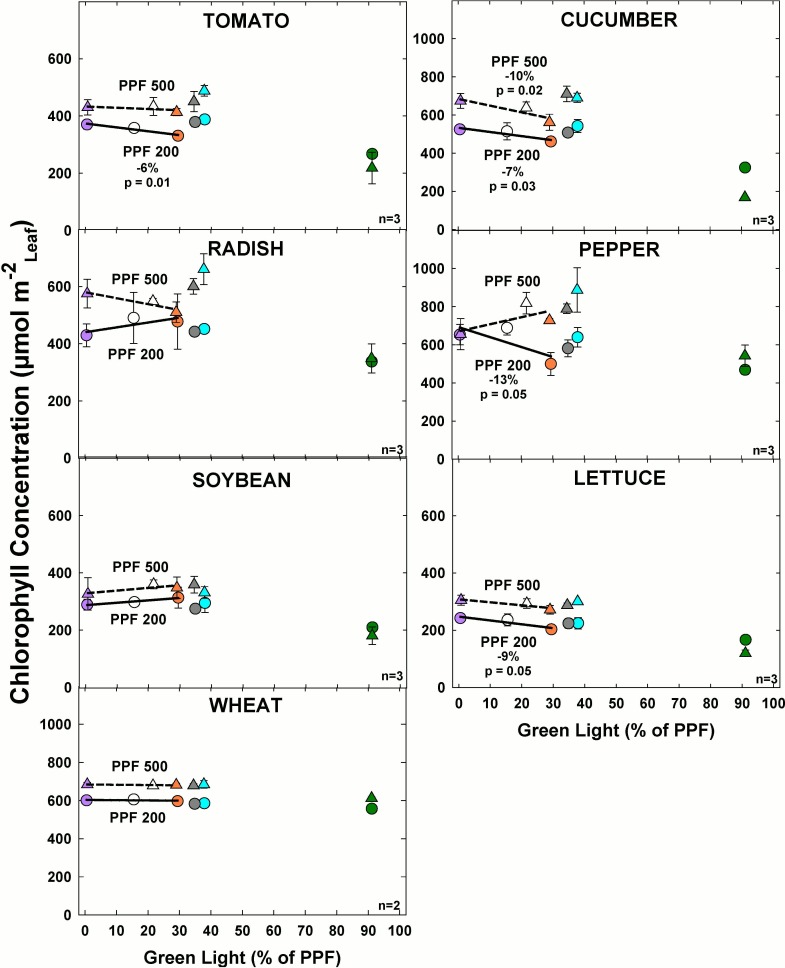
The effect of percent green light on chlorophyll concentration for seven species under two PPFs. Each data point shows the mean and standard deviation of three replicate studies for each species (n = 3). Some error bars are smaller than the symbol size. See Fig 1 for symbol color and shape legend. To minimize confounding spectral effects the regression line includes only the three treatments with comparable blue and red wavelengths for each PPF. When significant, p-values and percent change are shown.

Chlorophyll concentration significantly decreased with increasing GL only in cucumber at the higher light level among the comparable treatments (Fig 17). At the lower light level, chlorophyll concentration significantly decreased with increasing GL in tomato, cucumber, pepper and lettuce among the comparable treatments. For all other species at the lower light level there was minimal change in chlorophyll concentration and GL increased. Chlorophyll concentration increased with PPF for all species, but the magnitude of the change was not consistent among species.

## Discussion

### Effects of blue light

Although some BL is necessary for normal growth of many species, more BL is not necessarily better. Among the comparable treatments, the highest DM, greatest LAI, and longest stem and petiole length tended to occur in the lower BL treatments (≤11%). These results are consistent with the findings from several other studies on individual species and are consistent with a shade avoidance response in reduced BL.

Hernández and Kubota [12] found that leaf area and dry mass of cucumber decreased as BL increased up to 75%. Cope et al. [11], reported similar effects for lettuce, radish, and pepper, but they analyzed the effects of BL across all treatments (0 to 92% BL) including treatments that had potentially confounding factors, which might have caused growth effects associated with other variables.

Hoenecke et al. [18] grew lettuce seedlings for six days under treatments from 0 to 40% BL at two PPFs (150 and 300 μmol m^-2^ s^-1^) and reported that hypocotyl length rapidly decreased as BL increased. Brown et al. [52] reported that pepper stem length decreased as BL increased up to 21% BL. Dougher and Bugbee [15] examined the effects of BL on cell size and cell division and found that both were reduced under increased BL. Wargent et al. [53] found that UV radiation had a powerful inhibitory effect on leaf expansion in lettuce.

Monocots may have a fundamentally different response than dicot species. Dougher and Bugbee [6] found that BL had only a small effect on growth and morphology of wheat, and speculated that the response may be associated with the below ground meristem position of monocots during early growth. Our data for wheat are consistent with their results.

All of our spectral treatments were developed with LEDs, and this minimizes the confounding effects that occur when comparisons are made between LEDs and fluorescent lamps. The dry mass of all species under the three types of white LEDs was similar to the RB and RGB treatments. These results suggest that the beneficial effect of fluorescent light in previous studies could have been caused by factors other than spectral effects such as heat produced by far-red radiation [7,9,19,23].

### Net assimilation

The ratio of dry mass to leaf area index provides a measure of net assimilation integrated over time [42–45]. This integration interval is often one week but it the data can be integrated over longer time intervals. Several other studies have found that radiation capture, as predicted by LAI, is the dominant factor in determining growth rates [46–50]. When dry mass is expressed as a rate per day, the units for net assimilation also reflect a rate per day. Since the duration of these studies was constant for each species (21 days), growth and net assimilation values would be divided by a constant. The relative values are not changed dividing by the integration interval. It is possible that the ratio of growth to leaf area varied during the integration interval but integrating over the 21 day study provides an average value that can be compared among treatments. For consistency, results are shown for the entire 21 day integration interval. Because PPF was constant at either 200 or 500 μmol m^-2^ s^-1^, net assimilation rate provides a relative measure of photosynthetic efficiency. After the narrow band treatments are excluded, there was a surprisingly small effect of light quality on photosynthetic efficiency in any species at either PPF. When the spectral environment is consistent from day to day, leaf pigments may adapt to unique light environments over time.

This finding is in contrast to the effect of light quality on photosynthesis based on short-term, single-leaf measurements in several other studies. Hogewoning et al. [21], reported that increasing BL increased photosynthetic capacity (photosynthetic potential in higher PPF), but they used an unusually low baseline PPF of only 100 μmol m^-2^ s^-1^. Terfa et al. [22] found that increasing BL from 5 to 20% increased leaf thickness and increased photosynthetic capacity of single leaves. Hernández and Kubota [12] found that short-term net photosynthesis of single-leaves increased in cucumber as BL fraction increased from 10 to 80%, but whole-plant dry mass of the same plants steadily decreased as BL increased. These results highlight the often poor relationship between short-term single-leaf measurements [3] and whole plant dry mass gain.

The relative quantum efficiency of single leaves indicates that RL is 25 to 35% more efficient than BL and 5 to 30% more efficient than GL in driving photosynthesis [3,20]. However, this curve was measured at a low PPF over short intervals. Our results indicate that it is not appropriate to extrapolate from this curve to whole plants or plant communities grown at high PPF under mixed colors of light.

### Effect of self-shading on net assimilation

Net assimilation increased for some crops as BL increased. This is at least partly the result of self-shading. At canopy closure there is a reduction in the average PPF per leaf, which causes a reduction in the average net assimilation per leaf. In sensitive species, the lower BL treatments reached canopy closure sooner and had more self-shading. In contrast, the higher BL treatments reached canopy closure later and had less self-shading.

### Effects of green light

Remarkably, the dry mass of several species (peppers, wheat, low light cucumbers and soybeans) in narrow band GL was equal to the broad spectrum treatments and at the lower light level, increasing GL did not have a statistically significant effect on DM in any species. When all species are considered, we found only small effects of GL and the direction of the response was inconsistent between light levels.

In contrast to the findings of Wang and Folta [36], GL effects did not decrease as PPF increased. In tomatoes, radish and lettuce, the highest dry mass tended to occur in the lowest GL treatments but the effect was only statistically significant in radish at high light. Consistent with our results, Hernández and Kubota [12] found that increased GL (up to 28%) had no effect on cucumber dry mass. Kim et al. [54] reported that supplementing red and blue LEDs with green light (from green fluorescent lamps) increased lettuce dry mass by up to 48% at the same PPF but these results may be associated with the increase in diffuse light, or warmer leaf temperature, rather than a direct effect of GL.

Paradiso et al. [55] measured and modeled photosynthesis of individual rose leaves at 18 wavelengths and found increased utilization of GL in plant communities compared to individual leaves. These findings are consistent with those of Sun et al. [29] and Terashima et al. [31]. Collectively, these results indicate that measurements of spectral effects on single leaves in low light should not automatically be extrapolated to whole plant communities in higher light.

Increasing GL increased stem length in tomato, pepper, soybean, and lettuce, and increased petiole length in radish and soybean. Since GL is selectively enriched at the bottom of plant canopies, this result is consistent with a shade avoidance response, and the effect was greater at the lower PPF level. Increases in either stem or petiole length are typically associated with increased radiation capture and increased dry mass so increasing the fraction of GL might be used to enhance early growth in sensitive species. Interestingly, GL increased stem length in tomatoes but had no effect on petiole length.

In a comprehensive review, Folta and Maruhnich [35] suggested that GL can be a biological signal to slow down or stop growth. Our results indicate that the beneficial effects of GL on radiation capture offset any reductions in photosynthetic efficiency. It is important to note that harvest in these studies occurred shortly after canopy closure and the effects of GL on plant communities may be more beneficial in longer term studies as a greater fraction of the light is filtered to lower leaf layers.

### Shade avoidance responses for specific leaf area and leaf chlorophyll concentration

In addition to stem and petiole elongation, there were shade avoidance responses for leaf thickness (specific leaf area, SLA, m^2^_leaf_ per kg_leaf_ DM) (Figs 14 and 15). Increasing PPF increased leaf thickness (decreased SLA) in all species. Increasing BL tended to increase leaf thickness and the effect was statistically significant in cucumber, radish, pepper, and lettuce (Fig 14).

Increasing GL induced a shade avoidance response that tended to decrease leaf thickness (increased SLA) and the effect was statistically significant in cucumber, pepper, and soybean (Fig 15). Both BL and GL effects on specific leaf area are consistent with a shade avoidance response where more BL is associated with brighter sunlight, and more GL is associated with deeper shade.

There were shade avoidance responses for leaf chlorophyll concentration. Chlorophyll was significantly increased by higher PPF. Leaf chlorophyll concentration can sometimes increase in shade leaves when it is expressed on a per unit leaf mass basis. Our results are expressed per unit leaf area. Increasing BL also tended to increase leaf chlorophyll concentration and the effect was statistically significant in tomato, cucumber, radish, and pepper (Fig 16). Increasing GL tended to decrease chlorophyll concentration and the effect was statistically significant in tomato, cucumber, pepper and lettuce (Fig 17). These results are similar to those produced by Fan et al. [56]. Plants in low light conditions typically adapt by reducing chlorophyll concentration per unit leaf area [11].

### Anomaly in RB, low-PPF treatment for wheat

The wheat plants in the RB treatment at the lower light level had higher DM, LAI and net assimilation compared to the other treatments. This response was due to significantly increased tillering (data not shown). Tillering is presumably controlled by phytochrome responses, but the phytochrome photoequilibria was similar among chambers. The reason for increased tillering is unknown, but it occurred in each of the three replicate studies. There were no unique growth or developmental effects in this treatment for any of the other species. This response of wheat warrants further study.

## Conclusions

We have begun to characterize photobiological differences among species for both light quality and quantity. Dry mass of most, but not all, species was significantly better in the multiple wavelength treatments than in narrow band blue, green, or red light. Among the broad spectrum treatments at the higher PPF, increasing BL in four increments from 11 to 28% reduced dry mass in tomato, cucumber, and pepper by 22, 26, and 14% respectively, but there was no statistically significant effect on radish, soybean, lettuce and wheat. At the lower PPF, dry mass was reduced by 41% in tomato, but the effects of BL on the other species were less than 6% and were not statistically significant. Effects on leaf area paralleled effects on dry mass in all species at both PPFs, indicating that the effects of BL on dry mass were mediated by changes in leaf area. Since LAI is typically highly correlated with radiation capture and since radiation capture is typically highly correlated with dry mass gain, increases in radiation capture are likely responsible for nearly all of the increases in dry mass. Increased radiation capture can be the result of thinner, or less dense, leaves as indicated by a higher SLA. However, the relatively small changes in SLA that occurred were not adequate to explain the large differences in growth. This suggests that differences in growth were caused by altered carbon partitioning between leaves and stems, and more rapid coverage of the surface caused by longer petioles.

In contrast to the significant effect of BL on dry mass and leaf area, increasing GL from zero to 30% resulted in few significant differences on DM, LAI or net assimilation, and there was no consistent direction among species or PPF levels. Increasing GL increased stem and petiole length in several species, which is consistent with a shade avoidance response. Although GL had little effect on dry mass in these studies, its importance may increase over time as a dense canopy forms.

Revealing interactions between light quantity and quality was a primary goal of these studies. Previous studies have suggested that there is a minimum threshold for the number of blue photons for morphological responses. If this were the case, the responses should have been be greater at lower PPF. In contrast to this conclusion, these studies indicate that the effects of blue light were greater at the higher PPF. There was no evidence of a threshold for the response to blue light in any species.

Historically, studies to understand spectral effects on plant growth have often focused on single leaf photosynthetic efficiency. The results of this study suggest that these measurements do not provide a good indication of whole plant net assimilation rate in long-term studies.

Collectively, these results indicate that:

1. the effect of blue light on dry mass is primarily determined by changes in radiation capture and not by a direct effect on photosynthesis,
2. the effects of blue light fraction are greater at higher PPF,
3. there is a wide range in species sensitivity to blue light,
4. green light fraction has a minimal effect on dry mass gain during early growth,
5. the effects on leaf thickness and chlorophyll concentration in response to blue and green light fractions can be interpreted as a shade avoidance response,
6. light quantity can have a bigger effect on plant shape than light quality.
